# Nerve Decompression and Restless Legs Syndrome: A Retrospective Analysis

**DOI:** 10.3389/fneur.2017.00287

**Published:** 2017-07-06

**Authors:** James C. Anderson, Megan L. Fritz, John-Michael Benson, Brian L. Tracy

**Affiliations:** ^1^Anderson Podiatry Center for Nerve Pain, Fort Collins, CO, United States; ^2^Neuromuscular Function Lab, Department of Health and Exercise Science, Colorado State University, Fort Collins, CO, United States

**Keywords:** common fibular nerve, common peroneal nerve, peripheral neuropathy, Willis–Ekbom disease, nerve entrapment, surgical decompression

## Abstract

**Introduction:**

Restless legs syndrome (RLS) is a prevalent sleep disorder affecting quality of life and is often comorbid with other neurological diseases, including peripheral neuropathy. The mechanisms related to RLS symptoms remain unclear, and treatment options are often aimed at symptom relief rather than etiology. RLS may present in distinct phenotypes often described as “primary” vs. “secondary” RLS. Secondary RLS is often associated with peripheral neuropathy. Nerve decompression surgery of the common and superficial fibular nerves is used to treat peripheral neuropathy. Anecdotally, surgeons sometimes report improved RLS symptoms following nerve decompression for peripheral neuropathy. The purpose of this retrospective analysis was to quantify the change in symptoms commonly associated with RLS using visual analog scales (VAS).

**Methods:**

Forty-two patients completed VAS scales (0–10) for pain, burning, numbness, tingling, weakness, balance, tightness, aching, pulling, cramping, twitchy/jumpy, uneasy, creepy/crawly, and throbbing, both before and 15 weeks after surgical decompression.

**Results:**

Subjects reported significant improvement among all VAS categories, except for “pulling” (*P* = 0.14). The change in VAS following surgery was negatively correlated with the pre-surgery VAS for both the summed VAS (*r* = −0.58, *P* < 0.001) and the individual VAS scores (all *P* < 0.01), such that patients who reported the worst symptoms before surgery exhibited relatively greater reductions in symptoms after surgery.

**Conclusion:**

This is the first study to suggest improvement in RLS symptoms following surgical decompression of the common and superficial fibular nerves. Further investigation is needed to quantify improvement using RLS-specific metrics and sleep quality assessments.

## Introduction

### Background

Restless legs syndrome (RLS) was originally described by Willis in 1685 (1) and was then more fully medically characterized by Ekbom in 1950 (2). RLS is a major focus in the sleep clinic because it negatively impacts sleep, quality of life, and overall health (3, 4). The current diagnostic criteria for RLS include the urge to move accompanied by uncomfortable sensations during periods of rest that worsen in the evening (5). The attributes of the diagnostic criteria come from the Montreal Group, where they studied the suggested immobilization test. During an hour of voluntary immobilization, the subject is observed for periodic leg movements. Often the symptom intensity increases with increased duration of immobilization (6). These symptoms are often described by the subject as feeling, “Creepy-crawly,” “Jittery,” “Pulling,” “Shock-like pain,” “Burning,” “Throbbing,” “Tightness,” “Itching,” etc. (7), with pain being the primary symptom among over 50% of RLS patients (8). The sensations are usually experienced deep in the leg along with the corresponding sense of movements (7). The symptoms of RLS often degrade normal daily function but are improved with movement (5). RLS is prevalent in 5–10% of the general population (6), although studies have reported rates as high as 11% (9) and 15% (10).

### Diagnosis

Patients with RLS tend to seek medical care due to impaired sleep (11). The current diagnostic protocol relies primarily on subjective questionnaires because the physical and electrophysiological examinations typically appear normal in RLS. Therefore, the condition is often misdiagnosed. In fact, a study utilizing nerve conduction velocity testing found that there was no difference between those with and without RLS among those with diabetic peripheral neuropathy (12). Other conditions such as iron deficiency and comorbid neurological conditions, including peripheral neuropathy and radiculopathy, are commonly associated with RLS.

### Primary vs. Secondary RLS

The two commonly described forms of RLS are primary and secondary. Approximately two-thirds of RLS patients present with the primary form (13), which generally presents with an onset prior to 45 years of age and has a strong genetic/familial component (14, 15). The familial component is strong among those with idiopathic RLS but not among those with RLS and peripheral neuropathy (15) or secondary RLS.

When diagnosed later in life, RLS is often secondary to other neuropathic disorders, associated with a comorbid condition (16), and more symptomatic than those with primary RLS (17). Other neurological conditions often found in conjunction with RLS include Parkinson’s disease (18), multiple sclerosis (19), myelopathies (20), essential tremor (21), and peripheral neuropathy (16, 22, 23).

### RLS Treatment

Due to accompanying comorbidities, RLS is often misdiagnosed or treated merely for symptoms (24). Furthermore, successful treatment is complicated by a poor understanding of the etiology and mechanisms of RLS. Clinical evaluation should attempt to distinguish primary vs. secondary RLS.

Prescription treatment of the strongly familial primary RLS includes dopamine agonists [ropinirole, pramipexole (25), rotigotine (26), cabergoline, levodopa]; alpha 2 delta ligands (gabapentin enacarbil (27), pregabalin (28)), opioid agonists (oxycodone/naloxone), and iron. Additional physical treatment methods include near-infrared spectroscopy, pneumatic compression, transcranial direct current or magnetic stimulation, and vibrating pads.

Secondary RLS can be treated with prescription of ropinirole, levodopa, vitamin C and E supplementation, or exercise. However, this treatment strategy is only efficacious for those with end-stage renal disease on hemodialysis (24). End-stage renal disease can reduce erythropoietin levels. The resulting anemia can cause nerve damage and possibly induce secondary RLS.

#### Iron Supplementation

Iron is essential in the development of neuronal networks (29), synthesis, maintenance of myelin (30), and dopamine metabolism. Iron deficiency can inhibit vesicular neurotransmitter release (31) and reduce dopamine receptor density in various brain regions (32). In fact, low brain iron concentration is often found in RLS patients. Iron supplementation produces some treatment benefit in RLS, but not to the same extent as other drugs such as dopaminergic agonists (33). This may be explained by the finding that brain iron concentrations are dissociated from peripheral iron content, which suggests ineffective CNS iron acquisition mechanisms as a causative factor in RLS development (34). Furthermore, the expression of iron-management proteins is reduced in patients with RLS, which may contribute to decreased iron content (35).

Specifically, the substantia nigra often displays decreased iron content in RLS patients (36), and other studies support the idea of ineffective iron acquisition and decreased iron concentrations in those with RLS. It is also worth noting that oligodendrocytes, which produce myelin in the CNS, stain more intensely with iron than other brain cells (37). Therefore, the iron deficiency commonly found in RLS patients could also be related to decreased white matter and myelination and the comorbidity often found in RLS and multiple sclerosis (38).

#### Dopaminergic Agonists

Although imaging studies have failed to provide convincing and consistent evidence of dopamine irregularities in patients with RLS (39), the hypothesis that RLS is due to abnormal dopamine metabolism comes from the observation of rapid response to dopaminergic agents in the early treatment of the condition (40). One report showed that 53% of patients on ropinirole exhibited improved RLS symptoms compared with 40.9% for placebo (41). Ropinirole efficacy does not seem to be dependent on age of RLS onset (42), which may explain the common use for both primary and secondary RLS (24). Although efficacious, there are notable side effects to consider for ropinirole. One study found that ~17% of patients on ropinirole experienced mild and temporary adverse events (33). Furthermore, another report found that 3.5% of patients on ropinirole developed clinically significant augmentation, or worsening, of symptoms (43).

Pramipexole, another dopamine agonist, has been found effective in treating RLS. One study found that 50–77% of patients on pramipexole rated their symptoms as “better” or “much better” compared with 38% for placebo (44). However, another study found that 60.8% of those on pramipexole reported adverse events (45).

Both of the popular dopaminergic medications discussed above (pramipexole and ropinirole) have been linked to nausea, hypotension, episodes of psychosis, and obsessive compulsive behaviors (46, 47). The potential for augmentation of symptoms needs to be considered for all studied dopaminergic medications (48).

#### Alpha 2 Delta Ligands

This class of drug works by selectively inhibiting voltage-gated Ca^2+^ channels containing the subunit alpha 2 delta. Abnormalities in calcium channel expression or function are present in several clinical conditions, including neuropathic pain. Treatment of the pathological state of the Ca^2+^ channel is the accepted mechanism of action of pregabalin and gabapentin. Studies have found that pregabalin and gabapentin selectively bind to the alpha 2 delta-1 and alpha 2 delta-2 subunits and thus reestablish the function of voltage-gated Ca^2+^ channels (49).

Gabapentin, commonly prescribed for neuropathy, has been shown in one study to improve symptoms of RLS by 85%. However, this same study showed that 80% of subjects reported at least some negative effects such as somnolence (20%) and dizziness (12%) (50).

Pregabalin, marketed as Lyrica and primarily used for epilepsy and neuropathic pain, has been shown to also be efficacious in treating RLS. One study found that a 124 mg/day dose was 90% effective in treating RLS symptoms (28). However, other studies found pregabalin only 63–71% successful and reported adverse reactions such as unsteadiness, drowsiness, and headaches (46, 51).

Reports suggest that alpha 2 delta ligands such as gabapentin and pregabalin have been associated with other adverse events, including weight gain and peripheral edema. However, the most regularly reported adverse reactions are neuropsychiatric, including sedation, fatigue, dizziness, and ataxia (52).

#### Opioids

Opioids are found to be effective for some RLS-related symptoms such as poor quality and length of sleep (24). Specifically, prolonged-release oxycodone/naloxone has been implicated as an RLS treatment (24). Oxycodone is a semi-synthetic opioid found to yield analgesic effects *via* the binding of µ-opioid receptors on neurons (53). Opioids bind at both the presynaptic nerve terminal and the postsynaptic neuron. Analgesia, the primary intended action of opioids, results due to inhibition of neurotransmitter release in the presynaptic nerve terminal in primary afferent neurons. In addition to their neuronal effect, opioid agonists bind to µ-opioid receptors elsewhere in the body, including the gastrointestinal tract, which can result in opioid-induced constipation (54). Formulations of oxycodone with naloxone are intended to counteract this adverse event. Naloxone is an opioid antagonist with low systemic bioavailability. Therefore, when taken orally, it exerts its function primarily in the gastrointestinal tract and counteracts the constipating effects of oxycodone (55).

Oxycodone–naloxone therapy has been shown to be effective in the treatment of RLS. One study of 197 participants reported that 12 weeks of oxycodone–naloxone treatment reduced average International Restless Legs Syndrome (IRLS) scores by 16.5 points compared with placebo (9.4). This study then followed up patients treated with the opioid formula over the following 40 weeks in what they called the extension phase. During this phase, they increased the opioid dosage from 5 to 40 mg and observed an additional 5.4-point drop in IRLS scores. However, during the extension phase, 57% of participants had treatment-related adverse effects, with 2% categorized as serious. It is therefore advised that opioids, which have a high rate of AEs, risk of abuse, and augmentation (56), should only be used as a treatment for RLS if other treatment is ineffective (57).

### RLS and Peripheral Neuropathy

Reports have shown an increased prevalence of RLS among those with various forms of peripheral neuropathy, ranging from 5.2 (58) to 54% (59). A few early studies found no difference in prevalence between neuropathy patients and controls (60–62). However, this may be due to less strict identification criteria prior to the validation and wide acceptance of the IRLS Study Group rating scale (63) or differences in the specificity or heterogeneity of the neuropathy [i.e., general peripheral neuropathy vs. diabetic sensory polyneuropathy (DSPN)] (64). Peripheral neuropathy may be considered an umbrella term that covers many forms of paresthesias of different etiology. This includes DSPN, demyelinating neuropathy, inflammatory demyelinating polyradiculoneuropathy, Fabry’s disease with neuropathy, hereditary neuropathy, small fiber painful neuropathy, and neuropathy due to Charcot-Marie-Tooth disease, all of which have been reported in conjunction with RLS (12, 17, 65–68). DSPN, however, is most frequently associated with RLS (17).

One study reported a greater prevalence of RLS among diabetic patients (18–29%) compared with controls (6–7%) (13, 16). The presence of polyneuropathy was the only variable associating RLS with diabetes and not the metabolic attributes of diabetes (16). However, a follow-up case-controlled study found the prevalence of RLS in diabetics to be independent of peripheral neuropathy (69). RLS in diabetic neuropathy appears to be independent of iron levels (16, 62), sex, and duration of diabetes (16). Diabetic neuropathy is often associated with damage to the small nerve fibers, which led to speculation of a small fiber mechanism associated with DSPN and RLS (70). This small fiber involvement distinguishes “peripheral” RLS from “central” RLS (71), which is thought to have a dopaminergic mechanism (72). However, others hypothesized that diabetes may inhibit dopaminergic control at the spinal level *via* excitatory nociceptive inputs associated with peripheral neuropathy (16).

Reports also showed that 27% of RLS patients had underlying peripheral neuropathy, and of those 34% had primary RLS (59). Those with peripheral neuropathy and RLS have higher IRLS scores, greater symptom severity, and more disturbed sleep compared with those with primary RLS (17). Studies have found that 92% of those with idiopathic RLS without neuropathy had a family history of RLS as opposed to only 13% of RLS patients with neuropathy; therefore, there may be a difference in the etiologic nature of primary and secondary RLS (15).

Emerging research in surgical decompression has found that the symptoms associated with polyneuropathy may be reversible by decompressing focal nerve entrapment sites in the lower extremity. Entrapment neuropathies constitute around 30% of all diabetic neuropathies (73). Peripheral nerve entrapment is associated with nerve enlargement, whereby those with diabetic neuropathy have tibial nerve cross-sectional areas twice as large as those of diabetics without neuropathy and non-diabetic controls (74). The enlargement of the posterior tibial nerve has been shown to significantly improve following surgical decompression (75). Surgical decompression in the lower extremity is similar to that of the surgical decompression of the median nerve at the carpal tunnel, whereby the neural entrapment is relieved by release of the fascia constituting the nerve tunnel. Reports have described a disorder analogous to RLS in the upper extremity, whereby carpal tunnel resection either improved or resolved the restlessness of the upper extremity (76). This implies that there may be a peripheral nerve tunnel entrapment component to RLS symptoms.

## Original Research

Although RLS is well defined in the literature, there is no consensus on its etiology [see review in Ref. (5)]. Peripheral nerve entrapment has been previously proposed as an important component of RLS (77). Anecdotally, surgeons performing surgical decompression for peripheral neuropathy also observe patient reports of RLS improvement. Recent reports have also found RLS improvement following lumbosacral decompression (78) as well as analogous improvement in upper extremity symptoms following surgical decompression for carpal tunnel syndrome (76). The purpose of this retrospective analysis was to assess patient-reported changes in symptoms associated with peripheral neuropathy and RLS following surgical decompression of the common and superficial fibular nerves.

### Subjects

The subjects were included through a retrospective analysis of patients who underwent decompression of the common and superficial fibular nerves for either diabetic (*n* = 14) or non-diabetic (*n* = 28) peripheral neuropathy. All surgical procedures were performed by Dr. James Anderson, DPM, at Anderson Podiatry Center for Nerve Pain. Eleven men and 31 women were included. All of the subjects were diagnosed with RLS by a physician prior to surgery through patient history and clinical presentation. Patients were also queried on impaired sleep quality, daily fatigue, fall risk, and location of perceived symptoms during the initial clinical assessment. Muscle strength (manual muscle test), physician-observed gait quality, and proprioception (Romberg’s test) were assessed during the physical examination. The self-reported average age of the patients was 63 ± 11 years at the time of RLS diagnosis. Surgical eligibility for peripheral neuropathy was based on medical history and physical examination, including visual analog scales (VAS) >6 and positive Tinel’s sign in the affected nerve tunnels (79). See Anderson (80) for a detailed description of the clinical examination and surgical decompression.

Benefits, risks, and alternatives were presented, and informed consent was obtained from all subjects prior to surgery. Forty-two subjects underwent nerve decompression surgery between October 23, 2013, and December 9, 2015. The protocol and retrospective analysis were approved by the University of Colorado Health Institutional Review Board.

### Visual Analog Scale

Subjects scored their symptoms for the surgical leg on a scale of zero (no symptoms) to 10 (severe symptoms) without the use of medication. Scores were obtained during normal clinical visits both before surgery and 15 ± 5.6 weeks post-surgery for the following symptoms: pain, burning, numbness, tingling, weakness, balance, tightness, aching, pulling, cramping, twitchy/jumpy, uneasy, creepy/crawly, and throbbing.

### Surgical Decompression

The surgical decompression was performed in accordance with previously described methods for both the common (80) and superficial fibular nerves. Some subjects also underwent decompression of the deep fibular (57%), tibial nerve at the tarsal tunnel (14%), or tibial nerve at the soleal sling (57%) entrapment sites, depending on their clinical presentation.

### Statistical Analysis

Repeated measures analysis of variance was used to assess the differences between the pre- and postsurgical VAS ratings for each symptom. Total pre-surgery and total post-surgery VAS scores were calculated by summing the individual symptom categories. The change in total VAS was calculated (post-surgery minus pre-surgery). Correlations between pre-surgery VAS and the change in VAS were also calculated. To account for the multiple pre–post comparisons of symptoms, a more stringent *P* < 0.01 value was used to determine statistical significance. An additional analysis was conducted to account for the multiple comparisons using the Holm–Bonferroni Method (81).

### Results

**Figure 1:**
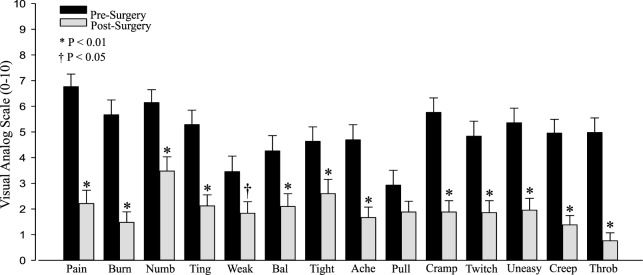
Pre- and postsurgical visual analog scale (VAS) by category: pain, burning (Burn), numbness (Numb), tingling (Ting), weakness (Weak), balance (Bal), tightness (Tight), aching (Ache), pulling (Pull), cramping (Cramp), twitchy/jumpy (Twitch), uneasy, creepy/crawly (Creep), and throbbing (Throb).

**Figure 2:**
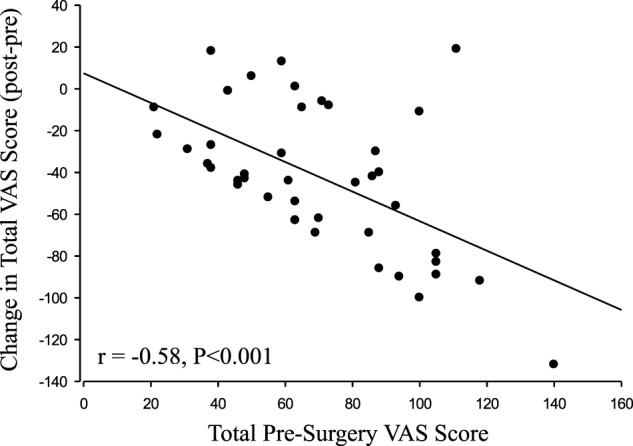
Change in visual analog scale (VAS) correlation (*r* = −0.58, *P* < 0.001).

All of the individual symptoms exhibited significant improvement following decompression (Figure 1), with the exception of “Pulling” (*P* = 0.14). The changes in the Weakness (*P* = 0.019) VAS score did not reach the *P* < 0.01 level of significance. The Holm–Bonferroni analysis produced *P*-value– ≤ 0.01 for all of the VAS scores, except Pulling (*P* = 0.05). The change in VAS score was negatively correlated with the pre-surgery VAS score for both the total summed VAS (*r* = −0.58, *P* < 0.001, Figure 2) and all of the individual symptom VAS scores (all *P* < 0.01).

There were no differences between men and women or between diabetics and non-diabetics for the change in VAS score after surgery (*P* > 0.05).

### Limitations

Due to the retrospective nature of this study, it did not include an assessment of other key attributes of RLS, such as iron deficiency. The relationship between RLS and iron deficiency remains unclear as some studies have found RLS to be independent of iron deficiency among diabetic patients (62), whereas others have found no statistical difference between hematocrit and serum iron and ferritin levels among those with primary RLS compared with neuropathy/secondary RLS (17).

### Conclusion

The main finding from this retrospective analysis is that surgical decompression of the common and superficial fibular nerves improved patient ratings of symptoms commonly associated with RLS. This preliminary finding suggests the need for rigorous, controlled, prospective research on the potential for nerve decompression surgery as a viable treatment option for RLS patients.

The total change in VAS was negatively correlated with the total presurgical VAS. Therefore, those who presented with the most severe symptoms experienced the greatest improvement following nerve decompression. This report is limited in that the data were collected retrospectively without a detailed collection of RLS-specific patient characteristics, including detailed differentiation between primary and secondary RLS. Nevertheless, these patients underwent decompression surgery for the treatment of peripheral neuropathy symptoms and also exhibited improvement in RLS-related symptoms, which is a finding that warrants additional investigation.

## Summary

Nerve decompression can reduce neuropathy symptoms for many diabetic and non-diabetic patients (82, 83). Symptoms specific to RLS, including burning, throbbing, creepy/crawly, and tingling (17, 84), also improve after decompression. Recently, surgical decompression for lumbosacral radiculopathy was found to improve RLS symptoms, sleep quality, depression, and fatigue (78). While the mechanisms of improved RLS symptoms remain unclear, the author speculated that reduced radiculopathy pain improved the central imbalance of dopamine and cytokines associated with RLS symptoms. However, they did not assess pain symptoms in their patients. Pain reduction may play a role in RLS improvement; however, it is unlikely to explain the dramatic decompression-related improvement in RLS symptoms due to the fact that the median pain level associated with RLS is only 18/100 on a VAS scale (85). The current understanding of RLS in terms of the mechanism of disease manifestation, diagnosis, treatment, and relationship to other comorbid neurological conditions remains unclear. A possible nerve compression contribution to the condition and symptoms may elucidate a decompression treatment option for individuals who do not experience relief from the current pharmaceutical interventions.

## Ethics Statement

This study was approved by the University of Colorado Health Institutional Review Board. Subjects gave informed consent prior to clinical treatment, however, they did not consent to the study as this is a retrospective analysis of de-identified clinical data and do not require informed consent for inclusion in the study.

## Author Contributions

JA performed the clinical examination and surgical decompression. J-MB and MF retrieved the subject clinical data from the health record archives. MF and BT performed the statistical analysis. All of the authors contributed to the composition and final approval of the manuscript document.

## Conflict of Interest Statement

JA owns Anderson Podiatry Center and receives income from his clinical practice in which nerve decompression surgery is one source of revenue. JA did not conduct or have any influence over the outcomes of this analysis as it was performed retrospectively and he did not participate in the collection of the VAS data, health record retrieval, or statistical analysis. The integrity of the research is bolstered by the collaboration with the Neuromuscular Function Lab at Colorado State University, which did not have any financial interest in the outcome of this study.
